# Assessment of Medication Dosage Adjustment in Hospitalized Patients With Chronic Kidney Disease

**DOI:** 10.7759/cureus.13449

**Published:** 2021-02-20

**Authors:** Zair Hassan, Iftikhar Ali, Arslan R Ullah, Raheel Ahmed, Adnan Zar, Irfan Ullah, Shakeel Rehman, Aziz Ullah Khan, Rizwan Ullah, Muhammad Hanif

**Affiliations:** 1 Cardiology, Lady Reading Hospital, Peshawar, PAK; 2 Pharmacy, Paraplegic Center, Peshawar, PAK; 3 Internal Medicine, Northwest General Hospital & Research Centre, Peshawar, PAK; 4 Nephrology, Institute of Kidney Diseases, Peshawar, PAK; 5 Internal Medicine, Lady Reading Hospital, Peshawar, PAK; 6 Internal Medicine, Kabir Medical College, Peshawar, PAK; 7 Pharmacy, Shaukat Khanum Memorial Cancer Hospital and Research Centre, Peshawar, PAK; 8 Pharmacy, Lady Reading hospital, Peshawar, PAK; 9 Internal Medicine, Khyber Medical College Peshawar, Hayatabad Medical Complex, Peshawar, PAK

**Keywords:** chronic kidney disease, renal impairment, medication errors, dose adjustment

## Abstract

Background

Inappropriate medication dosing can cause adverse drug reactions or ineffective therapy due to declined renal function in patients with renal insufficiency. This necessitates proper renal dose adjustment. This study was proposed to evaluate medication dosage adjustment in hospitalized chronic kidney disease (CKD) patients.

Methods

This study included all CKD patients hospitalized between May 1, 2019, and April 25, 2020, at the Institute of Kidney Disease, Peshawar, Pakistan. The estimated glomerular filtration rate was calculated using the Modification of Diet in Renal Disease formula, and dose appropriateness was established by evaluating practice with relevant reference books.

Results

Of the total 1,537 CKD patients, 231 (15.03%) had evidence of dosing error, which was considered for final analysis. Overall, 1,549 drugs were prescribed; 480 (30.99%) drugs required dose adjustment, of which 196 (40.42%) were adjusted properly and the remaining 286 (59.58%) were unadjusted. The most common unadjusted drugs were meropenem, cefepime, ciprofloxacin, and rosuvastatin, whereas captopril, aspirin, bisoprolol, pregabalin, and levofloxacin had the highest percentage of adjusted drugs. On multivariate logistic regression, the number of drugs requiring dosing adjustments and obstructive nephropathy were found to be statistically significant factors that increased the likelihood of the medication dosing errors: a unit increase in the number of drugs requiring dose adjustment increases 5.241 times the likelihood of dosing error. Similarly, the presence of obstructive nephropathy (OR: 0.383; 95% Cl: 0.153-0.960; p = 0.041) was found to be significantly associated with dosing error after adjustment for potential confounding factors.

Conclusion

The dosing of more than half of the prescribed drugs that required adjustment in CKD patients was not adjusted, which showed that medication dosing errors were high. This highlights the importance of medication prescription according to guidelines in these patients to improve the outcomes of pharmacotherapy.

## Introduction

Chronic kidney disease (CKD) is defined as “a decrease in glomerular filtration rate (GFR) <60mL/minute /1.73m^2^ for ≥3 months”, irrespective of the etiology, and is classified into five stages based on GFR. CKD affects over 10% of the people globally and is one of the major public health challenges [1,2]. Approximately 5 to 10 million deaths are attributed to kidney diseases every year [3]. The prevalence of CKD in Pakistan is high, affecting 12.5% (11.4%-13.8%) of the population [4].

Kidneys are key structures responsible for regulating homeostasis, acid-base equilibrium, and electrolytes. Most of the drugs, particularly water-soluble and their metabolites, are eliminated largely by the kidneys. Therefore, drug dosing depends largely on kidney function [5]. Maintaining normal kidney function is indeed necessary for several drugs and potential active metabolites to be metabolized and eliminated. Unlike in patients with healthy kidney function, renal impairment when doses are not reduced or renally adjusted can lead to adverse drug reactions (ADRs) [5,6]. Furthermore, to minimize ADRs, toxicities, and therapeutic failure, dosage modifications according to renal function in patients with renal failure need to be individualized. With a decrease in renal function, the pharmacokinetic parameters of several drugs are so adversely changed that the normal doses transform either to augmented or diminished [7-9]. In admitted patients, lethal or ineffective doses may increase hospital stay, treatment cost, and, accordingly, add extra burden on both the patients and the healthcare systems.

Renal injury is a known risk factor for adverse drug events (ADEs) but remains overlooked very often by healthcare professionals (HCPs) [10]. A study by Hug et al. revealed that 10% of patients with CKD experienced an ADE, and, out of these, 91% were considered preventable and 51% were serious [11]. A number of published articles have described considerable dose adjustment related difﬁculties and medication errors in CKD patients [11,12]. Poor understanding of the importance and optimization of medication dosages is often a cause of prescribing errors in patients with compromised renal function [13,14].

The most common dosing error while managing CKD patients is observed during antimicrobial use, requiring attention and adjustment in these patients depending on the estimated GFR (eGFR) of patients [14]. Studies from China showed that antibiotics-related dosage error in CKD patients were 38.8%-60.3% [14,15].

Literature supports that medication that required dose adjustment in CKD patients is not adjusted accurately in admitted patients. This practice is common in both developed and developing countries; during hospitalization, around 25%-77% of drugs are adjusted inappropriately [10,16]. A study in the Netherlands found that patients with advanced stages of CKD (III, IV) reveal a high prevalence of unadjusted dose prescription [17]. Similarly, a study in Australia also reported a high level of inappropriate prescription in elderly CKD patients with diabetes, with polypharmacy [18]. To address such an important problem, multiple research studies have been published globally to evaluate the dosing error pattern, although the subject is not thoroughly investigated particularly in developing countries.

A literature search showed very limited published material on this subject in Pakistan. As of now, no published studies were to our knowledge carried out in this part of the country (Peshawar). Considering this, the study was proposed to evaluate medication dosage adjustment and the factors associated with inappropriate renal dose adjustments in admitted CKD patients in Peshawar, Pakistan.

This article was previously “published” as a preprint (Hassan Z, Ali I, Ullah AR, Ahmed R, Rehman S, Khan A. Assessment of medication Dosage Adjustment in Hospitalized Patients with Chronic Kidney Disease. medRxiv. 2020).

## Materials and methods

Study setting

This retrospective study was conducted from May 1, 2019, and April 25, 2020, at the Nephrology Department of the Institute of Kidney Disease (IKD), Peshawar, Pakistan.

Study design and sampling procedure

Data were extracted from the medical records of CKD patients admitted to the nephrology ward in a structured format. All diagnosed cases of CKD receiving at least one drug requiring adjustment with a length of hospital stay greater than 24 hours and treatment under the supervision of consultant nephrologists were included. The eGFR was calculated using the “Modification of Diet in Renal Disease (MDRD)”. The Cockcroft-Gault formula was unable to be applied due to the absence of documented body weight in patient charts. The GFR staging was determined for the individual patients depending on their current condition agreeing to “Kidney Disease: Improving Global Outcomes” (KDIGO 2012) guidelines. The identified patients included those with GFR stages G3a (45-59 mL/min), G3b (30-44 mL/min), G4 (15-29 mL/min), and G5 (<15 mL/min).

Assessment of medication dosing errors

Unfortunately, due to the non-availability of any national drug formulary and drug dosing guidelines for CKD patients in Pakistan, we had to rely on some of the reputed references and dose adjustment guidelines for the individual drug doses assessment for appropriateness by comparing the observed practices.

Dosage appropriateness was based on comparing the practice with the established recommendation: “Drug Dosing Adjustments in Patients with Chronic Kidney Disease” published by the American Academy of Family Physicians [2] and “Drug Information Handbook, 25th edition” published by Lexicomp® [19]. The guidelines were selected in consultation with a consultant nephrologist.

Dose appropriateness was evaluated by a physician and hospital pharmacist. Prescribed doses that were in accordance with the recommended guidelines were regarded as adjusted. Nonetheless, inappropriately dosed or dose given recommended for patients with the normal renal function was categorized as not adjusted.

Data analysis

SPSS Version 20 (IBM Corp., Armonk, NY, USA) was used for data analysis. Descriptive statistics were used to present the results such as frequency and percentage for categorical data, while mean (SD) or median (IQR) was appropriate for numerical data. Logistic regression models were used to calculate the associated factors “All medication per patient were unadjusted” [(Yes/No)] as the main outcome measure that describes the medication error. Results were described as odds ratios (ORs) along with 95% Cl. Those variables (p < 0.2) in the univariate analysis were included in the multivariate statistics. A p-value of <0.05 was measured statistically significant.

## Results

During the study period, a total of 1,537 patients’ medical charts were reviewed. However, a total of 231 patients (15.03% of screened patients) were included in the final analysis. The demographics and clinical characteristics of patients are listed in Table 1. Of the total 231 patients, the majority were male (184 [79.7%]). The mean (SD) age of the patients was 46.14 (±15.90) years. The mean (SD) length of hospital stay was 3.97 (±1.96) days. The majority (209 [90.5%]) of the patients were in the G5 stage of CKD followed by 14 (6.1%) in G4. The mean (SD) of the medications prescribed was 6.7 (±1.33). Around 85.3% of patients were prescribed more than five medications. Similarly, the majority (95.23%) of the patient's medications list comprised antibiotics. Comorbidities were present in most of the patients (77.92%), and among them hypertension (148 [64%]), diabetes mellitus (57 [24.67%]), and obstructive nephropathy (36 [15.58%]) were on the top of the list.

**Table 1:** Demographic and clinical characteristics of the patients (N=231) GFR, glomerular filtration rate;eGFR, estimated glomerular filtration rate; BUN, blood urea nitrogen

Variable	N (%)
Age (years), mean (SD)	46.14 (±15.90)
Gender	
Male	184 (79.65)
Female	47 (20.35)
Length of hospital stay (days), mean (SD)	3.97 (±1.96)
GFR category	
G3b	8 (3.46)
G4	14 (6.06)
G5	209 (90.48)
eGFR (mL/min/1.73 m^2^), mean (SD)	8.07 (±7.8)
BUN (mg/dL), mean (SD)	205.29 (±93.02)
Serum creatinine (mg/dL), mean (SD)	10.33 (±5.43)
Potassium (mmol/L), mean (SD)	4.89 (±1.01)
Number of drug prescribed, mean (SD)	6.7 (±1.33)
≤5	34 (14.72)
>5	197 (85.28)
Antibiotics prescribed	
Yes	220 (95.24)
No	11 (4.76)
Comorbidities present	
Yes	180 (77.92)
No	51 (22.07)
Diabetes mellitus	
Yes	57 (24.68)
No	174 (75.32)
Hypertension	
Yes	148 (64.07)
No	43 (18.61)
Ischemic heart disease	
Yes	21 (9.09)
No	210 (90.91)
Urinary tract infection	
Yes	19 (8.23)
No	212 (91.77)
Hepatitis B	
Yes	09 (3.90)
No	222 (96.10)
Hepatitis C	
Yes	21 (9.09)
No	210 (90.91)
Obstructive nephropathy	
Yes	36 (15.58)
No	195 (84.42)

A total of 1,549 numbers of medications were prescribed to the study patients. Of the total prescribed, 480 (30.99%) required dosage adjustment. The majority of medications were unadjusted (59.58%), as depicted in Table 2.

**Table 2:** Number and mean or median of properly adjusted and unadjusted drugs prescribed

Variable	N	%	Mean (SD)	Median (IQR)
Total number of drugs prescribed	1,549	100	6.7 (±1.33)	
Total number of drug prescribed requiring adjustment	480/1,549	30.99	2.08 (±0.86)	
Total number of drugs properly adjusted	194/480	40.42		1 (0-3)
Number of drugs unadjusted	286/480	59.58		1 (0-3)

The descriptive statistics, as depicted in Table 3, showed that the most frequently unadjusted medications were meropenem (100%), cefepime (100%), ciprofloxacin (100%), rosuvastatin (100%), cefoperazone/sulbactam (91.33%), ranitidine (65.71%), and piperacillin/tazobactam (85.71%). The most properly adjusted medications were aspirin (100%), captopril (100%), bisoprolol (100%), pregabalin (100%), levofloxacin (100%), vancomycin (87.5%), domperidone (80.7%), cefotaxime (78.12%), furosemide (69%), sodium bicarbonate (53.65%), and spironolactone (50%).

**Table 3:** List of drugs needing adjustment, properly adjusted, or unadjusted

Drug name	Drugs needing adjustment, N	Drugs adjusted, N (%)	Drugs unadjusted, N (%)
Meropenem	17	-	17 (100)
Sodium bicarbonate	41	22 (53.66)	19 (46.34)
Domperidone	52	42 (80.77)	10 (19.23)
Cefoperazone/sulbactam	127	11 (8.66)	116 (91.34)
Ranitidine	70	24 (34.29)	46 (65.71)
Furosemide	55	38 (69.09)	17 (30.91)
Cefotaxime	32	25 (78.12)	7 (21.87)
Metoclopramide	6	1 (16.66)	5 (83.33)
Piperacillin/tazobactam	7	1 (14.28)	6 (85.71)
Tranxemic acid	3	1(33.33)	2 (66.66)
Vancomycin	8	7 (87.5)	1(12.5)
Captopril	3	3 (100)	-
Aspirin	6	6 (100)	-
Ciprofloxacin	3	-	3 (100)
Cefepime	27	-	27 (100)
Spironolactone	6	3 (50)	3 (50)
Rosuvastatin	3	-	3 (100)
Ramipril	2	1 (50)	1 (50)
Bisoprolol	2	2 (100)	-
Fluconazole	5	2 (40)	3 (60)
Pregabilin	2	2 (100)	-
Levofloxacin	2	2 (100)	-
Linezolid	1	1 (100)	-
	480	194 (40.42)	286 (59.58)

Of the total variables, GFR category G5 (OR: 0.340; 95% Cl: 0.121-0.955; p=0.041), the total number of drug prescribed (OR: 1.826; 95% Cl: 1.444-2.310; p≤0.01), and drug requiring dose adjustment (OR: 4.818; 95% Cl: 3.054-7.600; p<0.001) were noted to be significantly associated with dosing error on univariate analysis, as shown in Table 4. Multivariate analysis was run for the variables that were found significant (p≤0.2) on univariate analysis. An increase in one medication needing dose adjustment increase error by 5.241 times. Similarly, the presence of obstructive nephropathy (OR: 0.383; 95% Cl: 0.153-0.960; p=0.041) was found to be significantly associated with medication error after adjustment for potential confounding factors.

**Table 4:** Regression analysis of demographic and clinical variables with medication dosing errors cOR, crude odds ratio; BUN, blood urea nitrogen; GFR, glomerular filtration rate

Variable	All medications per patient were unadjusted		cOR [95% Cl]	p-Value
	No	Yes		
Age (years), mean	46.43	45.53	1.004 [0.988-1.021]	0.601
Gender, n (%)				
Male	100 (77.5)	84 (82.4)	0.739 [0.384-1.424]	0.366
Female	29 (22.5)	18 (17.6)	Reference	
Length of hospital stay (days), mean	3.94	3.99	0.989 [0.867-1.128]	0.865
Serum creatinine (mg/dL), mean	10.01	10. 75	0.975 [0.929-1.023]	0.303
BUN (mg/dL), mean	204.68	206.06	1.000 [0.997-1.003]	0.911
Serum potassium (mmol/L), mean	4.91	4.87	1.038 [0.802-1.344]	0.777
GFR category, n (%)				
G3b and G4	17 (13.2)	5 (4.9)	Reference	
G5	112 (86.8)	97 (95.1)	0.340 [0.121-0.955]	0.041
Total of drugs prescribed, mean	7.12	6.18	1.826 [1.444-2.310]	<0.001
Antibiotics prescribed, n (%)				
No	4 (3.1)	7 (6.9)	Reference	0.193
Yes	125 (96.9)	95 (93.1)	0.434 [0.124-1.527]	
Drugs requiring dose adjustment, mean	2.46	1.60	4.818 [3.054-7.600]	<0.001
Comorbidity present, n (%)				
Yes	98 (74.8)	84 (82.4)	Reference	
No	33 (25.2)	18 (17.6)	0.623 [0.327-1.188]	0.151
Diabetes mellitus, n (%)				
Yes	38 (29.5)	19 (18.6)	1.824 [0.976-3.411]	0.060
No	91 (70.5)	83 (81.4)	Reference	
Hypertension, n (%)				
Yes	79 (61.2)	69 (67.6)	0.756 [0.438-1.304]	0.314
No	50 (38.8)	33 (32.4)	Reference	
Ischemic heart disease, n (%)				
Yes	15(11.6)	6(5.9)	2.105 [0.786-5.637]	0.138
No	114 (88.4)	96 (94.1)	Reference	
Urinary tract infection, n (%)				
Yes	10 (7.8)	9 (8.8)	0.868 [0.339-2.224]	0.769
No	119 (92.2)	93 (91.2)	Reference	
Hepatitis C, n (%)				
Yes	11 (8.5)	10 (9.8)	0.858 [0.349-2.107]	0.738
No	118 (91.5)	92 (90.2)	Reference	
Hepatitis B, n (%)				
Yes	4 (3.1)	5 (4.9)	0.621 [0.162-2.374]	0.486
No	125 (96.9)	97 (95.1)	Reference	
Obstructive nephropathy, n (%)				
Yes	15 (11.6)	21 (20.6)	0.508 [0.247-1.044]	0.065
No	114 (88.4)	81 (79.4)	Reference	

## Discussion

CKD patients are the high-risk population for drug-related problems, and among them, medication dosing errors are the most prevalent [20,21]. A large number of published studies [14, 20-26] have determined the medication dosing error in these patients, but, unfortunately, few to none published studies were, to our knowledge, conducted in this part of the region.

This study revealed that a total of 1,549 drugs were prescribed to the patients, with a mean (SD) of 6.7 (±1.33). Of the total, 480 (30.99%) medication orders needed dose adjustment, of which around 40.42% were adjusted and the rest (59.58%) were unadjusted, almost similar to the finding of a study conducted in Bahawalpur, Pakistan, on CKD patients [21]. Our findings are slightly higher than those reported by Saad et al. in which 37% of the prescription order were adjusted adequately at two university hospitals in Lebanon [20].

In this study, the prevalence of renal medication dosing error was considerably lower as compared t previous reports from India (81%), Palestine (73.6%), South Africa (68%), and Lebanon (63%) [21,26,27]. The low percentage of medication errors in these CKD patients might be due to the reason that they received treatment from a trained nephrologist. On the other hand, result of dosing error was higher in our study compared to the studies completed in Indonesia (20.0%) and Nepal (13.5%) [22,25]. The proportion of dosing error in our study was also greater than the studies conducted in Saudi Arabia, Australia, and France, which were 53.1%, 44.8%, and 34.0%, respectively [13,23,24]. This suggests that either there is a variation in prescribing practices between prescribers or the pharmacy services in our setting are lacking compared to developed states. In countries such as Indonesia and Nepal, the lower prevalence of medication dosing errors could be attributed to the active involvement of the pharmacist in direct patient care. Altunbas et al. reported a considerably lower prevalence of medication dosing errors (12.6%); however, the authors explained that the majority of the study participants had renal impairment, thereby necessitating nephrology consultation and optimized drug regimens, which could limit the generalizability of study findings [28].

The economic burden of CKD and its associated complications increases the overall healthcare expenditures. Moreover, the early presentation of this chronic condition extends the burden to an even young population and in effect leads to further financial burdens [3]. The Pakistani healthcare system is resource-limited, with organizational mismanagement and lack of pharmacist in the multidisciplinary team and direct patient care making it more worst.

In developed nations, the active involvements of pharmacists coupled with computerized dosage optimization systems are primarily responsible for the appropriate medication therapy. Most automated dosage adjustment systems automatically alert the HCPs including physicians and pharmacists regarding renal function status and the need for dose optimization [13,26]. Therefore, lack of pharmacists in clinical settings, lack of national dosing formulary, and computerized dose adjustment programs in Pakistan lead to higher medication dosing error.

The unadjusted drug proportion was higher in patients with G5, where 97/209 drug entries were unadjusted despite the fact that G5 is a more advanced disease stage. A study conducted in Ethiopia reported that in patients with stage 5 CKD, 80% of the drugs were inappropriately adjusted [29]. A study by Saad et al. described that drugs prescribed in patients with stage 5 CKD, nearly 37% drug required dose adjustment [20].

Another important aspect in this study is the unusual age distribution where the patient’s mean age is 46.14±15.90 years, which is much different from the developed and even underdeveloped countries [13,22,23]. This difference might be due to the low expectancy of life in a normal healthy Pakistani, which is almost 65 years. The high prevalence of CKD in young adults in Pakistan is due to the high burden of diabetes mellitus, hypertension, and renal calculi, as evident in our study result as well [30].

While assessing the pattern of a medication dosing error, the majority of prescribed drugs were ordered without consulting dose adjustment guidelines. These include cephalosporin antibiotics (cefoperazone/sulbactam, cefotaxime, and cefepime), meropenem, sodium bicarbonate, ranitidine, metoclopramide, furosemide, spironolactone, and rosuvastatin. These findings are in agreement with earlier studies with the exception of vancomycin and the cardiac medication (aspirin, rosuvastatin, bisoprolol, and spironolactone), which were prescribed more appropriately in this study [26,29]. This observation shows that Pakistani physicians in public healthcare facilities are underestimating the adverse events associated with several drugs. For instance, antimicrobials such as aminoglycosides, vancomycin, and cephalosporins have the potential to induce nephrotoxicity [29].

Considering “unadjusted of all medication per patient” [Yes or No]” as a dependent variable, the results of logistic regression revealed that age, sex, length of hospital stay, antibiotics prescribed, serum creatinine, blood urea nitrogen, potassium, diabetes mellitus, hypertension, ischemic heart disease, urinary tract infection, hepatitis B and C, and obstructive nephropathy did not show statistical significance. Similar findings have been reported by previous studies [20,21,29]. However, GFR categories G5 (crude OR=0.340) had more unadjusted drugs compared to G3b and G4, whereas an increase in one medication needing dose adjustment increases error by 1.826 times. Similarly, the total number of drugs per patient that required dose adjustment was significantly associated with medication error on univariate analysis. The findings of our study are consistent with the previous report by Ahsan Saleem and Masood, in which end-stage CKD and the number of prescribed drugs were significant determinants of medication dosing errors [21]. In contrast, Getachew H et al. reported that severity of renal impairment, prescribed medications requiring dose optimization, and the number of prescribed medications per patient differ with the percentage of properly adjusted drugs per patient [29].

Medication dosing errors were not related to diabetes in this study, which is not in line with a study by Khanal et al. [18]. Furthermore, the presence of antimicrobial inpatient prescription was not a significant predictor, similarly reported by Saleem and Masood [21].

Multivariate analysis showed that a unit increase in the number of drugs requiring dose adjustment increases the chances of medication error by 5.241 times. Similarly, the presence of obstructive nephropathy (adjusted OR: 0.383) was found to be significantly associated with medication error after adjustment for potential confounding factors. Similar results have been described by a similar study where the numbers of prescribed medicines and the presence of comorbidities were significant determinants of medication dosing errors [21].

In CKD patients, the medications should be selected and prescribed with extreme precautions and appropriateness to avoid possible drug-related problems and adverse outcomes. The predictors identified should be paid attention to.

Strength and limitations

This study envisages several strengths. The first strength is that it is the first study performed in the tertiary care hospital of Peshawar, Khyber Pakhtunkhwa, and the second in Pakistan. Secondly, in comparison to the previous study, it is more detailed and looks into the pattern and determinants of medication dosing errors identified in admitted CKD patients. Despite the strength, the present study has several limitations. Firstly, our study was limited by small sample size. Secondly, a retrospective study design was used, which restricted us from suggesting interventions and observing actual ADRs. Thirdly, the MDRD formula was applied due to insufficient data on patient medical charts, such as weight, which is not suitable for higher muscle mass patients and those with malignant conditions such as cancer. Fourthly, the nephrologist may have consulted dose adjustment guidelines other than we used or all the patients may have not seen by the nephrologist. It is plausible to conclude that apart from renal function status, physicians may have based dosage optimization on blood pressure, serum electrolytes, and heart rate. ADEs in correlation to a medication error were not looked and neither therapy was followed. Finally, due to limited resources, the present study represented a single-center data and the finding cannot be generalized.

Clinical implications and future recommendations

An appraisal of the literature in correlation with the findings of the present study revealed that errors in medication dosing are a common clinical issue, particularly in patients with CKD. Dosing errors necessitate adequate attention by HCPs, and CKD patients need to be specifically evaluated for dosage optimization before prescribing medication. Moreover, there exists considerable confusion regarding the need and extent of dosage adjustment in patients with varying degrees of renal impairment; hence, harmonized and universally acceptable guidelines should be formulated and adopted regarding the dosage optimization of renally excreted drugs so as to safeguard the patient’s health. In developing countries including Pakistan, the active involvement of trained clinical pharmacists in direct patient care should be ensured in order to promote appropriate and optimized medication therapy. Furthermore, continuous medical educational programs, seminars, and workshops need to be organized on a regular basis regarding the optimization of medication therapy, particularly in patients with renal impairment. Therefore, intensiﬁed collaboration between HCPs (general practitioners, nephrologists, and clinical pharmacists) is recommended with the relevant exchange of patient information in a bid to reducing the rate of inappropriate prescription and medication dosing errors.

## Conclusions

The medication-related dosing errors were quite high; more than half of the drugs need a dose adjustment. The result also showed that appropriate dose adjustment for impaired kidney function was not accomplished in a large number of patients in the province's largest teaching hospital with specialist nephrologists and qualified residents who are expected to have a greater knowledge of dose adjustment in CKD patients. The determinants of medication associated dosing error in our study were the number of drugs requiring dose adjustment and obstructive nephropathy. Patients presenting with either obstructive nephropathy or several medications that require renal dose adjustment should have their medications closely scrutinized for renal dose adjustments.
